# A Novel Subunit Vaccine Based on Outer Capsid Proteins of Grass Carp Reovirus (GCRV) Provides Protective Immunity against GCRV Infection in Rare Minnow (*Gobiocypris rarus*)

**DOI:** 10.3390/pathogens9110945

**Published:** 2020-11-13

**Authors:** Changyong Mu, Vikram N. Vakharia, Yong Zhou, Nan Jiang, Wenzhi Liu, Yan Meng, Yiqun Li, Mingyang Xue, Jieming Zhang, Lingbing Zeng, Qiwang Zhong, Yuding Fan

**Affiliations:** 1 Yangtze River Fisheries Research Institute, Chinese Academy of Fishery Sciences, Wuhan 430223, China; muchangyong@yfi.ac.cn (C.M.); zhouy@yfi.ac.cn (Y.Z.); jn851027@yfi.ac.cn (N.J.); liuwenzhialisa@yfi.ac.cn (W.L.); mengy@yfi.ac.cn (Y.M.); liyq@yfi.ac.cn (Y.L.); xmy@yfi.ac.cn (M.X.); zhangjm@yfi.ac.cn (J.Z.); zlb@yfi.ac.cn (L.Z.); 2 College of Biological Science and Engineering, Jiangxi Agricultural University, Nanchang 330045, China; 3 Institute of Marine and Environmental Technology, University of Maryland Baltimore Country, Baltimore, MD 21202, USA; vakharia@umbc.edu

**Keywords:** grass carp reovirus, baculovirus expression system, subunit vaccine, immune response

## Abstract

The grass carp hemorrhagic disease, caused by the grass carp reovirus (GCRV), has resulted in severe economic losses in the aquaculture industry in China. VP4 and VP35 are outer capsid proteins of GCRV and can induce an immune response in the host. Here, three recombinant baculoviruses, AcMNPV-VP35, AcMNPV-VP4, and AcMNPV-VP35-VP4, were generated to express recombinant VP4 and VP35 proteins from GCRV type II in insect cells by using the Bac-to-Bac baculovirus expression system to create a novel subunit vaccine. The expression of recombinant VP35, VP4, and VP35-VP4 proteins in Sf-9 cells were confirmed by Western blotting and immunofluorescence. Recombinant VP35, VP4, and VP35-VP4 were purified from baculovirus-infected cell lysates and injected intraperitoneally (3 μg/fish) into the model rare minnow, *Gobiocypris rarus*. After 21 days, the immunized fish were challenged with virulent GCRV. Liver, spleen, and kidney samples were collected at different time intervals to evaluate the protective efficacy of the subunit vaccines. The mRNA expression levels of some immune-related genes detected by using quantitative real-time PCR (qRT-PCR) were significantly upregulated in the liver, spleen, and kidney, with higher expression levels in the VP35-VP4 group. The nonvaccinated fish group showed 100% mortality, whereas the VP35-VP4, VP4, and VP35 groups exhibited 67%, 60%, and 33% survival, respectively. In conclusion, our results revealed that recombinant VP35 and VP4 can induce immunity and protect against GCRV infection, with their combined use providing the best effect. Therefore, VP35 and VP4 proteins can be used as a novel subunit vaccine against GCRV infection.

## 1. Introduction

Grass carp (*Ctenopharyngodon idella*) is an important commercial species used in freshwater aquaculture in China [1]. In 2018, the annual production of grass carp in China was 5.50 million tons and accounted for 21.63% of the harvest in all freshwater fisheries. However, frequent outbreaks of hemorrhagic disease have become prevalent in many provinces of China, leading to huge losses in aquaculture [2]. In grass carp, hemorrhagic disease is caused by the grass carp reovirus (GCRV).

GCRV is a member of the genus *Aquareovirus* and family *Reoviridae*, and it was the first viral pathogen identified in aquatic animals in China in 1983 [3]. GCRV has an icosahedral symmetry, with an overall diameter of approximately 60–85 nm. It is composed of 11 segments of double-stranded RNA, which encode seven structural proteins (VP1–VP7) and five nonstructural proteins [4]. GCRV can be divided into three genotypes (GCRV I, II, and III) on the basis of genomic and biological characteristics, and GCRV II is responsible for the major pandemics of grass carp hemorrhagic disease in China. However, most of the studies have focused on GCRV I, and there is limited information on GCRV II in China [5].

Immunization is an effective method for the prevention of hemorrhagic disease. Currently, various types of GCRV vaccines are available, for example, inactivated, attenuated, subunit, and DNA vaccines. The inactivated and attenuated vaccines are easy to use and have the advantage of providing immunity to natural infections; however, these vaccines are unsafe and unstable, and it is easy to recover from mutations leading to disease [6]. DNA vaccines have advantages such as production ease, genetic stability, absence of cold chain requirement, and activation of humoral and cellular immunities. Currently, there is much debate on the safety of DNA vaccines because of the length of time the plasmid is continuously expressed in the fish; it is unclear whether the long-term expression of the plasmid in the body has adverse effects [7,8]. Subunit vaccines are typically prepared from viral capsid proteins and bacterial glycoproteins, and they have enhanced immune protection effects. When compared with the other vaccines, subunit vaccines can present an effective antigen epitope, stimulate the immune response without introducing pathogens, and can be produced as multivalent vaccines because of the low cost and safety [2].

The VP35 protein encoded by the S11 gene fragment of GCRV type II and VP4 protein (outer capsid protein) encoded by the S6 gene fragment [9,10] play an important role during virus entry into cells and can induce an immune response in the host [11,12], so they can be used as candidate protein vaccines [13]. Gao et al. (2018) expressed recombinant VP35 protein in prokaryotic cells and evaluated the immune protection of the recombinant VP35 protein by performing a series of experiments on grass carps. The results showed that the relative percentage of survival (RPS) was about 60% after the grass carps were challenged with GCRV. This indicates that the recombinant VP35 protein can protect grass carp from GCRV infection and be used as a subunit vaccine [14]. Jiang et al. (2018) used *Bacillus subtilis* spores as the oral delivery system and successfully constructed *B. subtilis* CotC-VP4 recombinant spores. The grass carps were orally immunized to assess protective efficacy, and a survival rate of 57% and RPS of 47% were recorded after the viral challenge [15].

However, it is difficult to use the grass carp as a model to study the immune protection of subunit vaccines against GCRV in vivo because of its large size and long reproductive cycle. Therefore, it is necessary to find a suitable fish model to evaluate the immune defense mechanisms underlying the response to reovirus infection [16]. The rare minnow, *Gobiocypris rarus*, is a species native to China, and it belongs to the family Cyprinidae; it can be infected with GCRV, which results in as high as 100% mortality [17]. In this study, we used the rare minnow as the model fish to study the mechanism underlying GCRV disease.

Baculovirus protein expression is based on eukaryotic expression systems; thus, baculoviruses exhibit protein modification and a processing pattern similar to those of higher eukaryotic cells [18]. The baculovirus expression system is safe because the host range is highly specific. Over the past 20 years, the baculovirus expression system has been widely used for the production of recombinant proteins and development of subunit vaccines in insect cells. In this study, we generated three subunit vaccines containing VP4, VP35, and VP35-VP4 of GCRV II via the baculovirus expression system and verified their immune protection against GCRV in the rare minnow.

## 2. Results

### 2.1. VP35 and VP4 Gene Cloning and Identification of Donor Vectors pFastBac-VP35, pFastBac-VP4, and pFastBac-VP35-T2A-VP4

**Figure 1:**
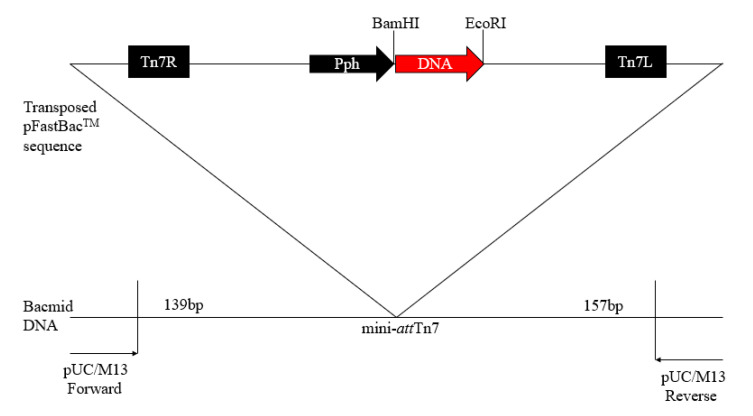
Schematic construction of the recombinant baculovirus.

**Figure 2:**
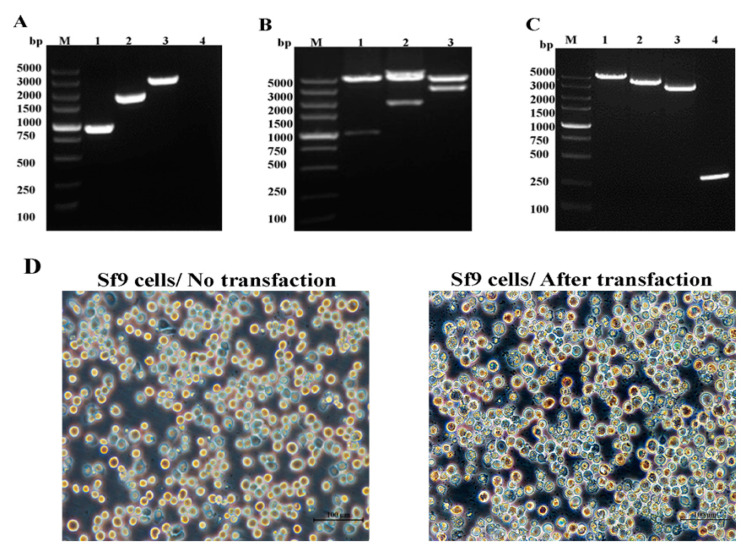
Construction of donor vectors pFastBac-VP35, pFastBac-VP4, and pFastBac-VP35-VP4 and generation of recombinant baculoviruses. (**A**) PCR amplification of VP35, VP4, and VP35-VP4: lane M, DNA marker; lane 1, VP35; lane 2, VP4; lane 3, VP35-VP4. (**B**) Analysis of recombinant plasmid: lane M, DNA marker; lanes 1–3, double-enzyme digestion of pFastBac-VP35, pFastBac-VP4, and pFastBac-VP35-VP4 with *Bam*HI and *Eco*RI. (**C**) Recombinant bacmid was confirmed using PCR analysis with M13. Lane M, DNA marker; lane 1, Bacmid- VP35-VP4; lane 2, Bacmid-VP4; lane 3, Bacmid-VP35; lane 4, Bacmid alone. (**D**) Sf-9 cells transfected with recombinant bacmid. The transfected cells typically exhibited an increase in cell diameter and nucleus size, ceased growth, lysis, and signs of clearing.

GCRV-106, VP4, VP35, and VP35-T2A-VP4 genes were cloned into pFastBac^TM^Iplasmids (Figure 1), using specific primers (F1/R1, F2/R2, F3/R3, and F4/R4, respectively) and detected with 1.5% agarose gel electrophoresis (Figure 2A). The VP35 gene is 933 bp in size and encodes a putative protein of 310 amino acid residues, and the VP4 gene is 1827 bp in size and encodes a putative protein of 606 amino acid residues. The donor plasmids pFastBac-VP35, pFastBac-VP4, and pFastBac-VP35-T2A-VP4 were digested with *Bam*H I/*Eco*R I, and the specific DNA bands were detected (Figure 2B). These plasmids were further characterized by sequencing the DNA.

### 2.2. Identification of Recombinant Baculoviruses

**Figure 3:**
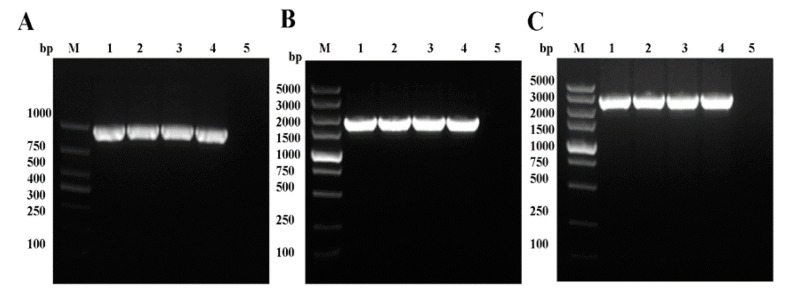
Identification of recombinant baculoviruses. The PCR products corresponding to VP35, VP4, and VP35-VP4 were amplified from the genome DNA of P1, P2, and P3 recombinant baculoviruses (AcMNPV-VP35, AcMNPV-VP4, and AcMNPV-VP35-VP4). Plasmids pFastBac-VP35, pFastBac-VP4, pFastBac-VP35- VP4, and pFastBac I were used as positive and negative controls. (**A**) PCR amplification of VP35 from AcMNPV-VP35: lane M, DNA marker; lanes 1–3, P1–P3 AcMNPV-VP35; lane 4, pFastBac-VP35; lane 5, pFastBac I. (**B**) PCR amplification of VP4 from AcMNPV-VP4: lane M, DNA marker; lanes 1–3, P1–P3 AcMNPV-VP4; lane 4, pFastBac-VP4; lane 5, pFastBac I. (**C**) PCR amplification of VP35-VP4 from AcMNPV-VP35-VP4: lane M, DNA marker; lanes 1–3, P1–P3 AcMNPV-VP35-VP4; lane 4, pFastBac-VP35-VP4; lane 5, pFastBac I.

The recombinant Bacmid-DNA was successfully generated (Figure 2C), and Bacmid-VP35, Bacmid-VP4, and Bacmid-VP35-T2A-VP4 were transfected into Sf-9 cells. Seventy-two hours after transfection, the Sf-9 cells showed enlarged nuclei and cessation of cell growth, granular appearance, lysis, and clearing of the monolayer (Figure 2D). The genomes of P1, P2, and P3 viruses were extracted and successfully confirmed using PCR with the primer pairs F1/R1 and F2/R2 (Figure 3A–C).

### 2.3. Expression of VP35 and VP4 Proteins in Sf-9 Cells

**Figure 4:**
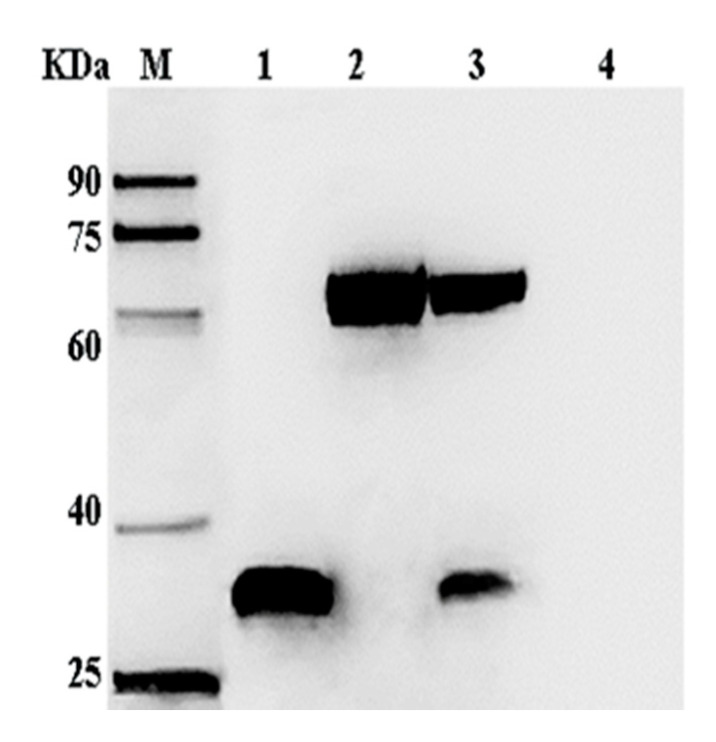
Western blotting analysis of VP35 and VP4 expression in Sf-9 cells. Lane M, protein markers; lanes 1–3, Sf-9 at 72 h post-infection with recombinant viruses AcMNPV-VP35, AcMNPV-VP4, and AcMNPV-VP35-VP4, respectively; lane 4, Sf-9 at 72 h post-infection with wild virus. The primary antibody was mouse anti-His-tag, and the secondary antibody was HRP-conjugated goat anti-mouse IgG.

**Figure 5:**
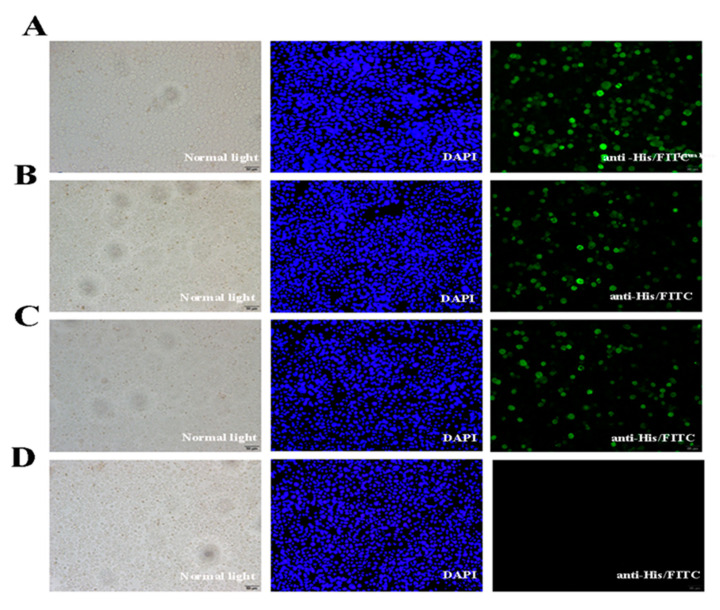
Immunofluorescence assay of recombinant protein expression in Sf-9 cells inoculated with the recombinant virus. (**A**–**D**) Sf-9 cells transfected with AcMNPV-VP35, AcMNPV-VP4, AcMNPV-VP35-VP4, and wild virus, respectively. From left to right, the Sf-9 cells under normal light, UV light, and treated with mouse anti-His-tag and Dylight 488-conjugated goat anti-mouse IgG.

The Sf-9 cells were infected with P3 baculovirus, collected after 72 h, and the proteins examined by SDS-PAGE and Western blotting. Protein bands of around 34 and 67 kDa that represented the recombinant VP35 and VP4 proteins, respectively, were visualized and identified by immunostaining (Figure 4). The immunofluorescence analysis showed that the VP35 and VP4 genes were expressed efficiently in the Sf-9 cells (Figure 5).

### 2.4. mRNA Expression of Immune-Related Genes in Different Tissues

**Figure 6:**
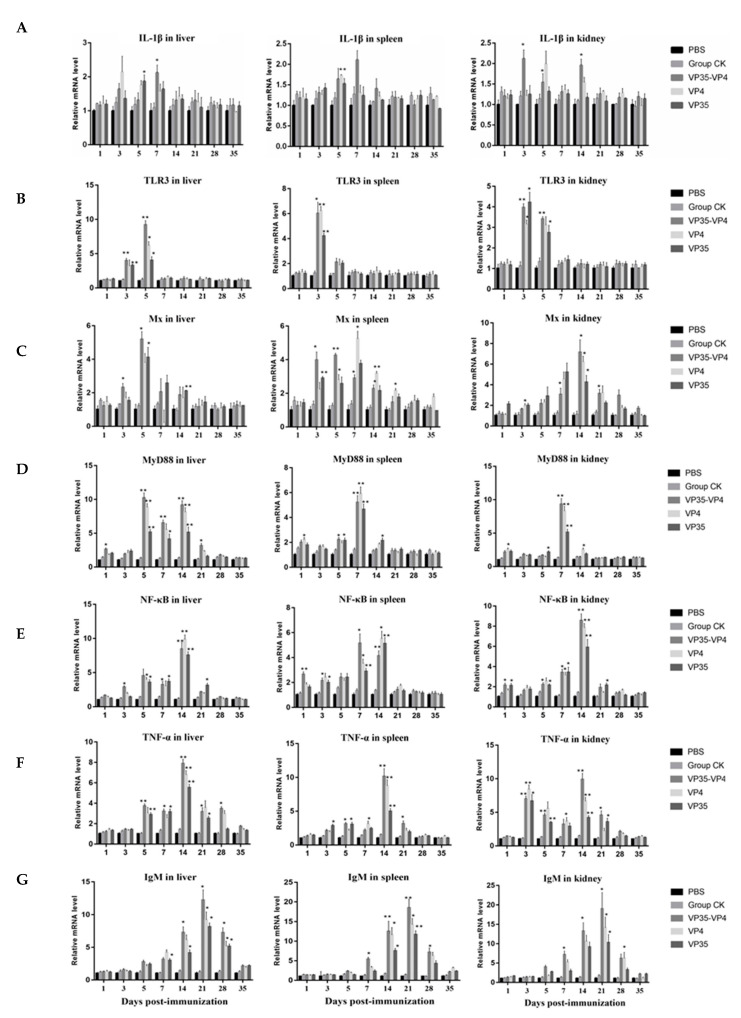
qRT-PCR analysis of the expression of immune-related genes in different tissues of the rare minnow on days 1, 3, 5, 7, 14, 21, 28, and 35 after immunization with different vaccines. (**A**) IL-1β, (**B**) TLR3, (**C**) Mx, (**D**) MyD88, (**E**) NF-κB, (**F**) TNF-α, (**G**) IgM. The mRNA level of each gene was normalized on the basis of β-actin gene expression. * denotes significant difference (*p* < 0.05), and ** denotes extremely significant difference (*p* < 0.01). The significant difference is compared against the group CK group. The relative mRNA expression levels of the immune-related genes were calculated using the 2^−ΔΔCt^ method.

The mRNA expression levels of immune-related genes IL-1β, TLR3, Mx, MyD88, NF-kB, TNF-α, and IgM were detected in the livers, spleens, and kidneys by using qRT-PCR. The relative expression levels of these genes were upregulated initially and then returned to normal in the immunized groups (Figure 6). The IL-1β expression levels of the VP35-VP4 group started to increase in the liver and kidneys at 3 days post-immunization (dpi) and reached peaked at 7 and 3 dpi (*p* < 0.05), respectively (Figure 6A). These results were 2- to 3-fold of the values obtained from the control CK. However, the IL-1β expression levels of the VP4 group peaked in the kidney, spleen, and liver at 3 dpi (1.9-fold, *p* < 0.05; 1.7-fold, *p* < 0.01) and 5 dpi (2.3-fold, *p* < 0.05), but no significant changes were detected in the control group. The TLR3 expression levels in the three immunized groups peaked in the spleen and kidneys at 3 dpi, and the levels in the VP35-VP4, VP4, and VP35 groups were about 6-fold (*p* < 0.05), 6.2-fold (*p* < 0.01), and 4.3-fold (*p* < 0.01), respectively, in the spleen and approximately 4-fold (*p* < 0.01), 3.3-fold (*p* < 0.05), and 4.1-fold (*p* < 0.05), respectively, in the kidney (Figure 6B). However, the TLR3 expression levels in the liver began to increase significantly at 3 dpi and peaked at 5 dpi: approximately 9-fold (VP35-VP4 group, *p* < 0.01), 7-fold (VP4 group, *p* < 0.05), and 4-fold (VP35 group, *p* < 0.05), respectively, that of the control. No significant differences in TLR3 gene expression were found in the PBS group when compared with the control CK group. In the liver, the relative expression levels of the Mx gene started to increase at 3 dpi and peaked at 5 dpi (*p* < 0.05). In the spleen, the relative expression levels of Mx progressively increased up to 21 dpi in the immunized groups and were significantly upregulated from 3 to 14 dpi (*p* < 0.05). In the kidney, the relative expression levels of Mx started to increase at 5 dpi and peaked at 14 dpi (*p* < 0.05), and the same trend was observed in all three immunized groups (Figure 6C). Moreover, the transcription levels of Mx were significantly higher in the VP35-VP4 group at 14 dpi than in the VP35 and VP4 groups.

The relative expression levels of MyD88 in the spleen and kidney of the three immunized groups (Figure 6D) were considerably upregulated at 1 dpi and peaked at 7 dpi (*p* < 0.01), and they were approximately 4–10-fold higher than the control. In the liver, the relative expression levels of MyD88 peaked at 5 dpi (*p* < 0.01) and 14 dpi (*p* < 0.01). The relative expression levels of NF-κB and TNF-α in the liver, spleen, and kidney of the three immunized groups (Figure 6E,F) increased at 5 dpi and peaked at 14 dpi (*p* < 0.01); the same trend was observed in all three immunized groups. The mRNA expression levels of IgM (Figure 6G) began to increase significantly at 7 dpi and peaked at 21 dpi, and the same trend was detected in the liver, spleen, and kidney of the three immunized groups. Specifically, in the VP35-VP4 group, the expression levels of IgM started to increase significantly at 5 dpi and peaked at 21 dpi in the liver, spleen, and kidney (12-fold, *p* < 0.05; 18-fold, *p* < 0.01; and 19-fold, *p* < 0.05, respectively). Similarly, in the VP4 and VP35 groups, the expression levels of IgM began to increase significantly at 7 dpi and peaked at 21 dpi in the liver (9-fold, *p* < 0.05; 8-fold, *p* < 0.05, respectively), spleen (15-fold, *p* < 0.05; 13-fold, *p* < 0.01, respectively), and kidney (14-fold, *p* < 0.05; 11-fold, *p* < 0.05, respectively). The transcription levels of IgM were significantly higher in the VP35-VP4 group at 7–28 dpi than in the VP4 and VP35 groups.

### 2.5. Challenge Test

**Figure 7:**
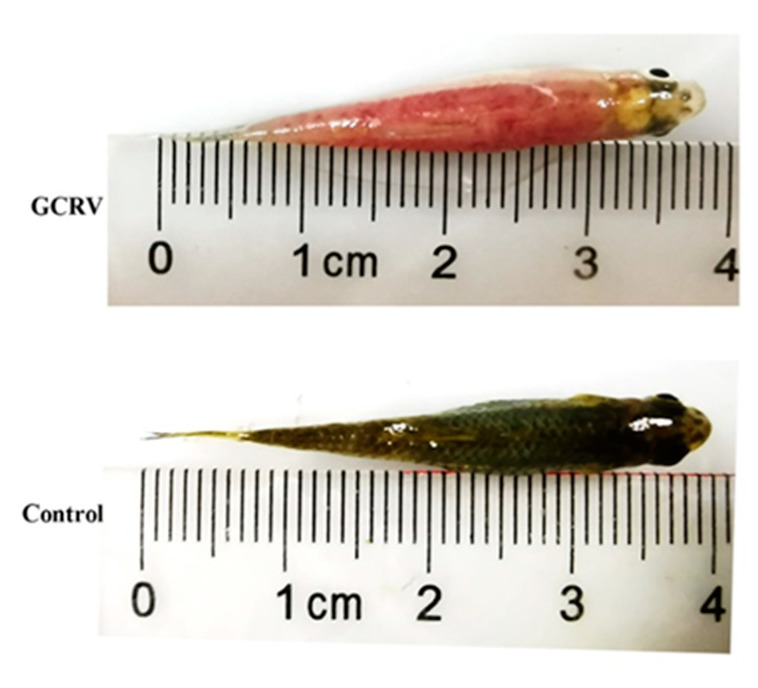
Hemorrhagic symptoms induced by GCRV-106. Images show representative fish specimens in the GCRV-106 and control groups, with the former exhibiting typical muscular hemorrhagic symptoms.

**Figure 8:**
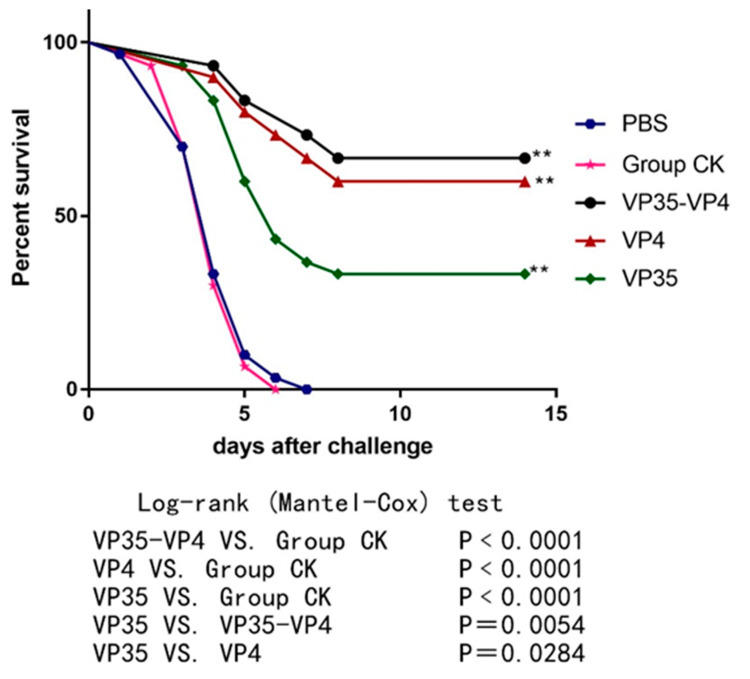
Cumulative survival percentage of the experimental fish challenged with the virus. ** denotes extremely significant difference (*p* < 0.01).

To evaluate the immune protective effects of the three vaccines, the fish were immunized with VP35-VP4, VP4, and VP35 and challenged with 10 μL of 1 × 10^5^ LD_50_ GCRV at 21 dpi. The mortality and clinical signs of the challenged fish were recorded daily for 2 weeks. The fish that died after infection with GCRV-II showed muscular hemorrhage throughout the body (Figure 7), and the virus was detectable using RT-PCR in the diseased fish. The cumulative mortality was 100% in the control CK and PBS groups after infection with GCRV (Figure 8). However, the RPS values of VP35-VP4, VP4, and VP35 groups were 67% (*p* < 0.01), 60% (*p* < 0.01), and 33% (*p* < 0.01), respectively. This suggests that VP35-VP4, VP4, and VP35 vaccines increased the survival rate of the rare minnows against GCRV. The VP35-VP4 group showed a higher protection rate than the VP35 group (67% vs. 33%, *p* = 0.0054), demonstrating that the VP35-VP4 vaccine can better protect the fish from hemorrhagic disease.

## 3. Discussion

The baculovirus expression system has been widely used as a powerful expression vector for the production of recombinant proteins and development of subunit vaccines in insect cells because of its biological safety, nonreplicative nature, low cytotoxicity, and large capacity [19]. For example, Yang et al. (2015) expressed the recombinant VP60 protein in a baculovirus system and induced high HI titers that rendered complete protection in guinea pigs [20]. Li et al. (2011) produced a foot-and-mouth disease subunit vaccine for cattle by using a silkworm–baculovirus expression system; the protective efficacy of the vaccine was found to be good via injection [21]. Musthaq et al. (2009) constructed a recombinant baculovirus with an immediate early promoter that expressed VP28 at an early stage of infection in insect cells, and they used this baculovirus as a vaccine against the white spot syndrome virus (WSSV) and recorded a significantly higher survival rate of 86.3% and 73.5% in WSSV-infected shrimp at 3 and 15 days post-vaccination, respectively [22]. Li et al. (2019) constructed a live vector vaccine based on the recombinant baculovirus BacCarassius-D4ORFs containing a fused codon-optimized sequence D4ORFs and relative immune protective rate; oral administration and abdominal injection with BacCarassius-D4ORFs resulted in a relative survival rate of 59.3% and 80.1%, respectively, in gibel carps, suggesting that BacCarassius-D4ORFs is a potential candidate vaccine against cyprinid herpesvirus II infection [23]. Expression of GCRV VP6 and VP7 proteins with the baculovirus expression system has shown that baculovirus vector vaccines can induce grass carp to produce an immune response, so such vaccines can be used as GCRV vaccine candidates [24,25]. Recombinant baculoviruses have been widely used as efficient tools to mediate gene delivery into mammalian cells, but they have rarely been used in fish cells [26]. 

BLAST analysis showed that the amino acid sequence of GCRV-GD108 VP4 is homologous to that of the structural protein VP4 in known aquareoviruses (27.3–32.9%). Immunogenicity prediction with the DNA Star software revealed multiple B-cell epitopes on GCRV-GD108 VP4, and the M6 gene has been speculated to encode the major outer capsid protein VP4. A neutralization assay demonstrated that the rabbit polyclonal antibody of rVP4 can prevent virus infection efficiently. Previous immunization experiments have shown that rVP4 provides protection against virus infection (47–82%), and rVP4 induces a high antibody titer in immunized fish [24]. The S11 segment in GCRV II encodes the VP35 protein, which has a conserved putative zinc-binding motif (CxxC-n16-HxC) analogous to that of MRV r3 [9,27,28]. Therefore, VP35 is considered as an outer clamp protein. Previous studies have shown that VP35 encoded by the S11 fragment appears to have good immunogenicity as both a DNA vaccine and subunit vaccine, and the survival rate after vaccination increased significantly (70.4–73.3% and 60%, respectively) [14,29]. Outer capsid proteins are conservative and play an important role in viral invasion and replication as key antigens [11,12,13,29]. Therefore, VP35 and VP4 can be used as candidate vaccines.

In the present study, we constructed recombinant baculoviruses AcMNPV-VP35, AcMNPV-VP4, and AcMNPV-VP35-VP4 and investigated their effective protective immunity against GCRV. To overcome the limitations of the restricted immune response induced by subunit vaccines that express a single antigen, VP35 and VP4 were linked with the self-cut T2A peptide to obtain simultaneous expression of two proteins in a vector and generate a subunit vaccine construct containing two antigens. 2A oligopeptides are autonomous elements containing a D(V/I)EXNPGP motif at the C terminus [30]. The small 2A peptide sequences, when cloned between genes, result in the generation of two individual proteins from an open reading frame (ORF) within a single vector through a “stop-carry on” recoding within the 2A peptide sequence, and this is termed as a novel “cleavage” event [31]. The 2A reaction occurs in the ribosomal peptidyltransferase center. Ribosomes pause at the end of the 2A coding sequence, over the glycine and proline codons, and the nascent chain up to and including this glycine is released [30]. It is possible to coexpress up to four full-length antigens joined by 2A sequences in an ORF under the control of a single promoter [32]. The use of the 2A peptide sequence avoids protein expression imbalance and large size when both genes are simultaneously expressed. Importantly, separation of genes placed between 2A peptide sequences is nearly 100% and allows for stoichiometric and concordant expression of the genes, regardless of the order of placement within the vector [31]. Mice vaccinated with V-2A developed antigen-specific cellular and humoral responses against all three antigens [32].

The rare minnow, a cyprinid species with a relatively small size (5–6 cm) and short reproductive cycle (3–4 months) [16], is a useful fish model. It is very sensitive to GCRV, and its mortality is 100% [17]. Therefore, the rare minnow has been selected as a model fish to study the protective effects of subunit vaccines against viral fish diseases. The VP33 protein has been expressed and purified to generate an anti-VP33 antiserum. The death of infected fish was delayed, and the mortality decreased to 10% when the fish were treated with the anti-VP33 antiserum, suggesting that it may be useful for the prevention and control of fish retroviral diseases [9].

Teleost fish have both innate and adaptive immunity. Innate immunity constitutes the first line of defense after pathogen invasion and plays an important role in immune defense [14,33]. The expression of immune-related genes could reflect the immune response level of fish [34]. Vaccines induce the relative mRNA expression of some important immune-related genes and enhance the immune ability of fish [35]. The thymus, kidney (anterior and middle), and spleen are the largest lymphoid organs in teleosts [36]. Therefore, in the present study, the relative mRNA expression levels of some immune-related genes in the liver, spleen, and kidney were measured using qPCR to evaluate the immune protection elicited by the vaccines.

In cells, TNF-α and IL-1β are two types of potent proinflammatory cytokines that play important roles in immunity and inflammation and in the control of cell proliferation, differentiation, and apoptosis [37]. Significant upregulation of IL-1β and TNF-α mRNA expression levels were found in spleen of grass carp at 6 weeks post VP4 protein immunization [15]. In this study, the TNF-α and IL-1β transcript levels increased significantly from 7 to 21 dpi in the immunized groups. This suggests that monocytes and macrophages may have a strong inflammatory response to antigens that enter the fish, which is necessary to enhance the immunity of the fish and ultimately eliminate foreign pathogens. The TLR-NF-κB pathway plays an important role in regulating the innate immune response, which is involved in the clearance of inducible and released inflammatory factors, to resist the invasion of pathogenic microorganisms [38,39]. TLR3, a member of the TLR family, is an indispensable recognition receptor for host defense against viral infection [40], and it was the first reported antiviral TLR [41]. In the current study, the expression of TLR3 in the spleen and kidney was significantly upregulated and peaked at 3 dpi. However, in the liver, the mRNA expression of TLR3 began to increase significantly at 3 dpi, peaked at 5 dpi, and it was the highest in the liver. TLR3 is responsible for recognizing dsRNA and initiating the immune response against dsRNA viruses [42]. The expression of TLR3 was upregulated in two strains of previously unexposed channel catfish in 2, 5, 8, and 21 days after challenge with *Edwardsiella ictaluri* [43]. This suggests that TLRs are important components of the innate immune system in catfish. Previous studies have shown that the transcription level of TLR3 was much higher (*p* < 0.05) in the liver of rare minnow after infection with GCRV than in the other tissues, and TLR3 regulates Mx expression and plays a crucial role in anti-GCRV immunity in the rare minnow by reducing the mortality and virus yield [44], suggesting that TLR3 may play an important role in fish defense against viral infection. 

Mx proteins are some of the most studied antiviral proteins induced by type I interferons in vertebrates, and they belong to a family of large GTPases [45]. A unique property of Mx GTPases is their antiviral activity against a wide range of RNA viruses; the antiviral activity of Mx was thought to be mediated by preventing the transport and replication of the viral nucleocapsid protein [46]. Chen et al. (2018) found that the Mx1 transcript in the pC-S6 group was upregulated at 7 dpi in the spleen and muscle of grass carp [7]. In our study, significantly higher expression levels of Mx were found in the liver, spleen, and kidney of the three immunized fish groups. Larsen et al. (2004) found that the Atlantic salmon Mx1 protein (ASMx1) possesses antiviral activity against the infectious pancreatic necrosis virus [47]. The expression of rainbow trout Mx can be induced by polycytidylic acid and chum salmon reovirus in trout monocyte/macrophage and fibroblast cell lines [48]. Previous studies have found that the rare minnow has Mx gene(s), but the introduction of more Mx genes improves its resistance to GCRV. Mx-transgenic rare minnows may contribute to the control of GCRV diseases [44]. Myeloid differentiation factor 88 (MyD88) is an important adaptor protein in the TLR signaling pathways, and the activation of MyD88 plays a central role in the innate immune system and contributes to the defense against invading pathogens [49]. In the present study, the relative expression levels of MyD88 was upregulated initially in spleen and kidney tissues and then returned to normal in the immunized groups. Therefore, we speculate that the activation of MyD88 in immune organs and tissues of immunized fish would be beneficial in resisting GCRV infection. 

Immunoglobulins are mainly produced by plasmablasts and plasma cells; they are secreted into body fluids as antibodies or found on the surface of B-cells as B-cell receptors and thought to be the only ones that respond to pathogens in both systemic and mucosal compartments [50]. Jiang et al. (2019) showed that specific IgM levels in the serum of grass carp increased from week 2 and reached its peak at week 4 after immunization with VP4 protein [15]. The expression levels of IgM in the spleen and kidney of grass carp in the VP35-immunized group increased at 7 dpi, attained a peak in the head kidney at 35 dpi, and reached the highest levels in the spleen at 28 dpi [29]. In our study, 1 week after the fish were treated with the subunit vaccines, the IgM expression levels in the liver, spleen, and kidney significantly increased and peaked at 21 dpi. The IgM expression levels were higher in the liver, spleen, and kidney in the VP35-VP4 group than in the other two groups. Previous studies have shown a significant increase in the expression of IgM after GCRV infection in the rare minnow, and the “Influenza A pathway” plays a critical role in the defense mechanisms against GCRV in the rare minnow; this is consistent with the results reported in grass carp [16,51]. Ou et al. (2019) showed that the expression of GrSRB1 in the main immune tissues of the rare minnow was upregulated after GCRV infection; this suggests that GrSRB1 is associated with GCRV and could bring GCRV to the cells, which facilitates virus attachment and entry [52]. We speculate that the immune responses of the rare minnow and grass carp are similar, and rare minnows are highly sensitive to GCRV. Therefore, the rare minnow can be used as a model fish to study the mechanisms underlying GCRV disease.

The results of the challenge experiment showed that the RPS values of VP35-VP4, VP4, and VP35 groups were 67% (*p* < 0.01), 60% (*p* < 0.01), and 33% (*p* < 0.01), respectively, and the VP35-VP4 group showed a higher protection rate than the VP35 group (67% vs. 33%, *p* = 0.0054); these results are consistent with the nonspecific immune response and immune-related gene expression levels. Therefore, the VP35-VP4, VP4, and VP35 subunit vaccines of GCRV elicit protection against GCRV infection in the rare minnow, with VP35-VP4 providing better protection than the other two vaccines.

In conclusion, our results revealed that recombinant VP35 and VP4 subunit vaccine can induce immunity and protect against GCRV infection, with their combined use providing the best effect. The interaction of VP35 and VP4 and their roles in GCRV invasion need to be studied further.

## 4. Materials and Methods

### 4.1. Ethical Statement

The study was performed in strict accordance with the Guide for the Care and Use of Laboratory Animals Monitoring Committee of Hubei Province, China, and the protocols (approval number: YFI2019fanyuding-01) were approved by the Committee on the Ethics of Animal Experiments at the Yangtze River Fisheries Research Institute, Chinese Academy of Fishery Sciences.

### 4.2. Materials

Healthy full-sib adult rare minnows (age, 3 months; weight, 1–1.5 g; average length, 5 cm) were obtained from the Institute of Hydrobiology, Chinese Academy of Sciences, Hubei, China. Prior to experimentation, fish were acclimatized at 28 °C for 2 weeks and then randomly sampled from the liver, spleen, and kidney for the detection of GCRV by RT-PCR to ensure that these fish were free of this virus. During the experimental manipulation, fish were kept at 28 °C and fed daily with a commercial diet. GCRV-106 (GCRV II) was isolated from diseased grass carps with severe hemorrhagic disease and maintained in our laboratory. Sf-9 cells were maintained at 28 °C in Grace’s Insect Medium containing 10% (*v*/*v*) FBS (Gibco, Gaithersburg, MD, USA). The expression vector pFastBac^TM^I and cell transfection reagent Cellfectin^TM^II Reagent were purchased from Thermo Fisher (Waltham, MA, USA). The pMD™19-T vector and competent *Escherichia coli* DH5α were purchased from TaKaRa (Dalian, China).

### 4.3. Gene Cloning and Analysis

The total RNA of GCRV-106 was extracted using TRIzol reagent. First-strand cDNA was synthesized using the PrimeScript^TM^ 1st strand cDNA Synthesis Kit (TaKaRa). The VP35 gene of GCRV-106 was amplified via PCR with the following primers: F1, 5′-GGGATCCATGGAACCAGCAAAACCATTGACG-3′ (the underlined nucleotides indicate the *Bam*HI site), and R1, 5′-GGAATTCTTAGTGATGGTG GTGATGATGCTGTCCCTGGATCTCAGGTTTG-3′ (the underlined nucleotides indicate the *Eco*RI site). The VP4 gene of GCRV-106 was amplified via PCR with the following primers: F2, 5′-CGGGATCCA TGCCGAATGGACTCGCA TGGATGAAAG-3′, and R2, 5′-CGAATTCCTTAGTGA TGGTGGTGATGATGAGA CGGAGGAGGCCAGTATCGAC-3′ (the underlined nucleotides in the forward and reverse primers indicate *Bam*HI and *Eco*RI sites, respectively), and 6 His tags were added to each protein. The PCR product was examined via electrophoresis with a 1.5% agarose gel and purified using the Wizard^@^ SV Gel and PCR Clean-Up System (Promega, Madison, WI, USA). The target DNA was ligated into the pMD19T vector (TaKaRa) to obtain plasmids pMD19T-VP35 and pMD19T-VP4. The recombinant plasmids were analyzed with enzyme digestion and sequencing. In addition, we constructed the fusion gene VP35-T2A-VP4 by using overlap extension PCR technology (SOEPCR), and VP35 and VP4 were joined with a self-cut T2A peptide. We redesigned two pairs of primers to amplify VP35 and VP4. The VP35 gene was amplified using the primer pair F3, 5′-GGGATCCATGGAACCAGCAAAACCATTGACG-3′ (the underlined nucleotides indicate the *Bam*HI site), and R3, 5′-AGCTAGCGTGATGGTGGTGA TGATGCATGCCGGAGCCGGACTGTCCCTGGATCTCAGGTTTGAAG-3′ (the underlined nucleotides indicate the *Nhe*I site). The VP4 gene was amplified using the primer pair F4, 5′-A GCTAGCCCGAATGGACTCGCATGGATGAAAG-3′, and R4, 5′-GGAATTCTCTAGTGATGGTGGTGATGATGAGACGGAGGAGGCCAGTATCGAGTTAATTTGT-3′ (the underlined nucleotides in the forward and reverse primers indicate the *Nhe*I and *Eco*RI sites, respectively), and 6 His tags were added to each protein. After two rounds of PCR, the amplified fragment was inserted into a pMD-19T vector (TaKaRa) to obtain the plasmid pMD19T-VP35-T2A-VP4. Then, the recombinant plasmids were sequenced.

### 4.4. Construction of Donor Vectors pFastBac-VP35, pFastBac-VP4, and pFastBac-VP35-VP4

VP35, VP4, and VP35-VP4 excised from pMD19T-VP35, pMD19T-VP4, and pMD19T-VP35-VP4 by digestion with *Bam*HI/*Eco*RI (New England Biolabs, Ipswich, MA, USA) were ligated into pFastBac^TM^I (Invitrogen, Carlsbad, CA, USA) to generate pFastBac-VP35, pFastBac-VP4, and pFastBac-VP35-VP4, respectively. The positive clones were extracted and confirmed by sequencing and enzymatic digestion with *Bam*HI and *Eco*RI. These plasmids were also verified using PCR with the following primers: pFastBac-F, 5′-TATTCCGGATT ATTCATACC-3′, and pFastBac-R, 5′-ACAAATGTGGTATGGC TGA-3′.

### 4.5. Generation of Recombinant Baculoviruses

According to the manufacturer’s instructions, pFastBac-VP35, pFastBac-VP4, and pFastBac-VP35-VP4 were transformed into *E. coli* DH10Bac/AcMNPV (Invitrogen, Carlsbad, CA, USA) to generate recombinant Bacmid-VP35, Bacmid-VP4, and Bacmid-VP35-VP4, respectively, by using the Bac-To-Bac baculovirus expression system (Invitrogen, Carlsbad, CA, USA). The recombinant bacmid DNA is greater than 135 kb in size. As restriction analysis is difficult to perform with DNA of this size, the PUC/M13 Forward primer, 5′-CCCAGTCACGACGTTGTAAAACG-3′, and Reverse primer, 5′-AGCGGATAACAATTTCACACAGG-3′, were used to verify the presence of Bacmid-VP35, Bacmid-VP4, and Bacmid-VP35-VP4 in the recombinant bacmid. The three Bacmid-DNAs were transfected into Sf-9 cells to generate the recombinant baculoviruses AcMNPV-VP35, AcMNPV-VP4, and AcMNPV-VP35-VP4 with Cellfectin^TM^II transfection reagent (Invitrogen, Carlsbad, CA, USA). The Sf-9 cells appeared lysed 72 h after transfection, and the viruses were collected from the cell culture and stored at 4 °C as the P1 viral stock and continuously proliferated through further infection of Sf-9 cells until the P3 viral stock was obtained and maintained at 4 °C in the dark. The P3 virus was identified using PCR with primer pairs F1/R1 and F2/R2.

### 4.6. Analysis of VP4 and VP35 Expression in Sf-9 Cells with Western Blotting

To verify protein expression, the harvested Sf-9 cells infected with recombinant AcMNPV at 72 h post-infection were subjected to SDS-PAGE analysis and Western blotting. Uninfected Sf-9 cells, which were similarly treated, were used as the negative control. Western blotting was performed with a mouse anti-His-tag (1:1000 diluted with TBST; Abcam, Cambridge, UK) overnight at 4 °C. The secondary antibody was alkaline phosphatase-conjugated goat anti-mouse IgG (1:5000 diluted with TBST; Thermo Fisher, Waltham, MA, USA) and visualized using the enhanced chemiluminescence method.

### 4.7. Detection of VP4 and VP35 Expression in Sf-9 Cells with Immunofluorescence Analysis

To confirm the expression of VP4 and VP35 proteins, Sf-9 cells were seeded on coverslips in 12-well plates and infected with recombinant AcMNPV (MOI 1) at 80% cell confluence. On day 3 post-infection, the cells were fixed with 4% paraformaldehyde for 20 min, rinsed three times with PBS, permeated with 0.2% Triton X-100 in PBS for 10 min at room temperature (RT), washed four times with PBS (5 min each with gentle shaking), and blocked with 5% BSA in PBS for 1 h at RT. The cells were incubated with mouse anti-His-tag (1:500 diluted with PBS) overnight at 4 °C, washed (five times, 5 min/time) with PBST, and then incubated with Invitrogen Alexa Fluor Plus 488 goat anti-mouse IgG secondary antibody (Thermo Fisher, Waltham, MA, USA) at a dilution of 1:1000 for 1 h at RT. After the cells were washed with PBST, the nuclei were stained with DAPI (Thermo Fisher, Waltham, MA, USA) for 15 min at RT. The fluorescent images were captured using a Leica DM2500 fluorescent microscope with Leica DFC420C camera (Leica, Wetzlar, Germany). 

### 4.8. Immune Protection and Sample Collection

The rare minnows were divided into experimental and control groups. The experimental group was randomly divided into three parallel groups (VP35, VP4, and VP35-VP4) with 60 fish per group, and the control group was randomly divided into two parallel groups (control CK and PBS) with 60 fish per group. In the three immunized groups, each fish in each group was injected intraperitoneally with 3 μg of recombinant VP35 (rVP35), rVP4, or rVP35-VP4 protein at a volume of 50 μL. In the control CK group, each fish was intraperitoneally injected with the same volume of Sf-9 cell lysate with the wild-type virus AcNPV. In the PBS group, each fish was intraperitoneally injected with the same volume of PBS. The experimental fish were maintained at 25 ± 2 °C and fed once daily. This experiment was performed three times independently. The liver, spleen, and kidney tissues were sampled from three fish at 1, 3, 5, 7, 14, 21, 28, and 35 dpi. Tissues for RNA extraction were placed in a DEPC-treated homogenate tube with 1 mL of TRIzol reagent and stored at −80 °C.

### 4.9. Detection of mRNA Expression of Immune-Related Genes by Using qRT-PCR

The total RNA was extracted from the liver, spleen, and kidney tissues of the fish by using TRIzol reagent, according to the manufacturer’s instructions. First-strand cDNA was synthesized using the PrimeScript™ 1st strand cDNA Synthesis Kit (TaKaRa). TB Green^®^ Premix Ex Taq™ II (TaKaRa) and real-time (RT)-PCR system (Bio-Rad, Hercules, CA, USA) were used to perform qRT-PCR and monitor the expression levels of genes involved in the immune response (IgM, TNF-α, MyD88, NF-κB, IL-1β, Mx, and TLR3). The gene expression levels were normalized using the housekeeping gene β-actin. The primers of the genes have been described in previous studies [16,44,52,53,54,55,56]. All samples from the immunized and control groups were tested in triplicate by using qRT-PCR. The PCR cycling conditions were as follows: one cycle at 95 °C for 30 s, followed by 40 cycles at 95 °C for 5 s and 60 °C for 30 s. The relative mRNA expression levels of the immune-related genes were calculated using the 2^−ΔΔCt^ method. 

### 4.10. GCRV Challenge Test

On 21 dpi, fish of the immunized and control groups (*n* = 30, each group) were intraperitoneally injected with 10 μL of 1 × 10^5^ LD_50_ GCRVII. The infected fish were maintained at 28 °C, and the other culture conditions were identical. The fish mortality was monitored daily for 14 days, and the dead fish were examined using RT-PCR to confirm that the fish died of GCRV infection. RPS was calculated as RPS = [1 − (% mortality of immunized group/% mortality of control group)] × 100%.

### 4.11. Statistical Analysis

Data were presented as mean ± standard deviation (SD). The statistical significance between the experimental group and the control group was analyzed by using ANOVA and Tukey’s tests in SPSS software (version 20.0). Differences were considered significant at *p* < 0.05 and highly significant at *p* < 0.01.
